# Angiography-derived index of microcirculatory resistance as a novel, pressure-wire-free tool to assess coronary microcirculation in ST elevation myocardial infarction

**DOI:** 10.1007/s10554-020-01831-7

**Published:** 2020-05-14

**Authors:** Giovanni Luigi De Maria, Roberto Scarsini, Mayooran Shanmuganathan, Rafail A. Kotronias, Dimitrios Terentes-Printzios, Alessandra Borlotti, Jeremy P. Langrish, Andrew J. Lucking, Robin P. Choudhury, Rajesh Kharbanda, Vanessa M. Ferreira, Oxford Acute Myocardial Infarction (OXAMI) Study Investigators, Keith M. Channon, Hector M. Garcia-Garcia, Adrian P. Banning

**Affiliations:** 1 Oxford Heart Centre, NIHR Biomedical Research Centre, Oxford University Hospitals, Headley Way, Oxford, OX39DU UK; 2 Oxford Centre for Clinical Magnetic Resonance Research (OCMR), University of Oxford, Oxford, UK; 3 MedStar Washington Hospital Center, Washington, DC USA

**Keywords:** Index of microcirculatory resistance, Microvascular obstruction, Quantitative flow ratio, Microvascular dysfunction, STEMI

## Abstract

Immediate assessment of coronary microcirculation during treatment of ST elevation myocardial infarction (STEMI) may facilitate patient stratification for targeted treatment algorithms. Use of pressure-wire to measure the index of microcirculatory resistance (IMR) is possible but has inevitable practical restrictions. We aimed to develop and validate angiography-derived index of microcirculatory resistance (IMR_angio_) as a novel and pressure-wire-free index to facilitate assessment of the coronary microcirculation. 45 STEMI patients treated with primary percutaneous coronary intervention (pPCI) were enrolled. Immediately before stenting and at completion of pPCI, IMR was measured within the infarct related artery (IRA). At the same time points, 2 angiographic views were acquired during hyperaemia to measure quantitative flow ratio (QFR) from which IMR_angio_ was derived. In a subset of 15 patients both IMR and IMR_angio_ were also measured in the non-IRA. Patients underwent cardiovascular magnetic resonance imaging (CMR) at 48 h for assessment of microvascular obstruction (MVO). IMR_angio_ and IMR were significantly correlated (ρ: 0.85, p < 0.001). Both IMR and IMR_angio_ were higher in the IRA rather than in the non-IRA (p = 0.01 and p = 0.006, respectively) and were higher in patients with evidence of clinically significant MVO (> 1.55% of left ventricular mass) (p = 0.03 and p = 0.005, respectively). Post-pPCI IMR_angio_ presented and area under the curve (AUC) of 0.96 (CI95% 0.92–1.00, p < 0.001) for prediction of post-pPCI IMR > 40U and of 0.81 (CI95% 0.65–0.97, p < 0.001) for MVO > 1.55%. IMR_angio_ is a promising tool for the assessment of coronary microcirculation. Assessment of IMR without the use of a pressure-wire may enable more rapid, convenient and cost-effective assessment of coronary microvascular function.

**Electronic supplementary material:**

The online version of this article (10.1007/s10554-020-01831-7) contains supplementary material, which is available to authorized users.

## Introduction

Coronary microvascular injury remains an important determinant of poor prognosis and an unsolved challenge in the management of patients with ST elevation myocardial infarction (STEMI). The index of microcirculatory resistance (IMR) has been proposed to provide information about the status of coronary microvasculature and it is based on the combined application of thermodilution technique and of coronary pressure-wire [1]. It has been validated against cardiovascular magnetic resonance imaging (CMR) [2] and against major clinical outcomes. Measured at the completion of the procedure, a post-pPCI IMR ≥ 40U is associated with a higher rate of mortality and readmission for heart failure in STEMI patients [3]. Moreover, IMR has been showed to provide information about the status of the microvasculature before stenting [4], and either alone or in combination with other clinical and anatomical parameters it can provide an immediate indicator of patients at high risk of suboptimal reperfusion [5, 6].

Despite encouraging preliminary results of studies showing the potential efficacy of IMR-guidance in triaging novel therapies in STEMI [7], IMR is still perceived as a research tool and its application within clinical practice remains extremely limited. Probable reasons for a lack of clinical penetration include the additional procedural time /complexity, increased procedural cost and the potential challenge of pressure wire manipulation in the infarct related artery (IRA) in STEMI patients.

Quantitative flow ratio (QFR) is a novel angiography-based index derived from application of computational flow dynamics to three-dimensional modelling of the coronary artery [8]. QFR has been shown to have a good correlation with invasive fractional flow reserve (FFR) and it appears to be superior to angiography in assessing the ischemic potential of angiographically intermediate coronary stenosis [9]. QFR does not rely on pressure-wire use, but it remains an index for characterization of coronary epicardial segment and does not provide direct assessment of coronary microcirculation.

By measuring QFR in the IRA, we aimed to derive and validate a novel index, the angiography-derived index of microcirculatory resistance (IMR_angio_), to provide a pressure-wire-free alternative to IMR for the assessment of coronary microvasculature.

## Methods

Patients with STEMI admitted to the Oxford Heart Centre for pPCI between September 2018 and August 2019 were prospectively considered for enrolment in the OxAMI (Oxford Acute Myocardial Infarction) study. Details about OxAMI study have been previously described [10]. The OxAMI study protocol was approved by the local ethics committee (REC number 10/H0408/24) and conducted in accordance with the Declaration of Helsinki.

STEMI was defined as the occurrence of ongoing chest pain for at least 30 min associated with ST-segment elevation > 2 mm in at least two contiguous leads. Enrolled patients were excluded for IMR_angio_ and IMR assessment in case of haemodynamic instability, evidence of angiographic left main disease, anticipated plain old balloon angioplasty without stent implantation or unsuitability for CMR assessment.

**Fig. 1:**
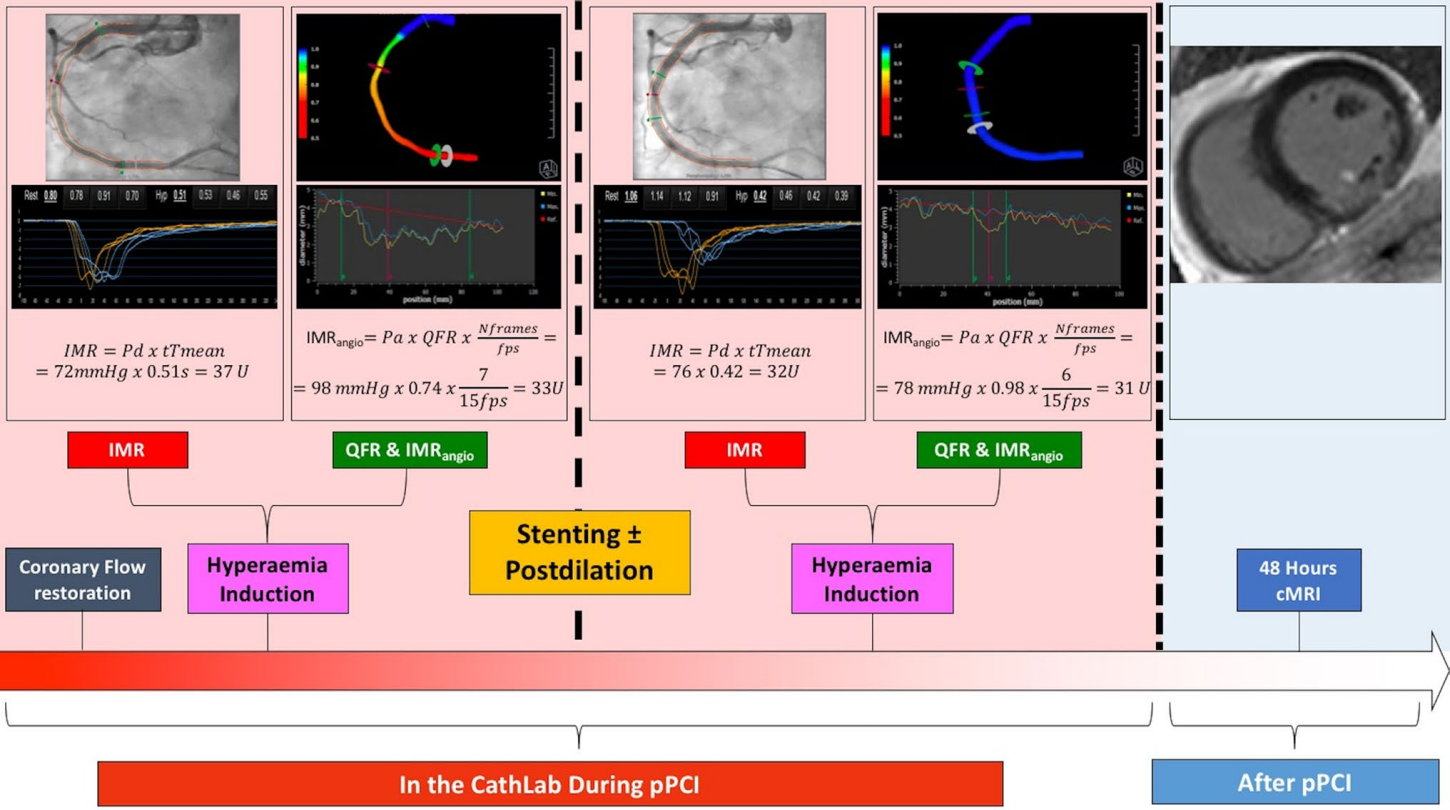
Study methods flow chart

Figure 1 summarizes the study-methods as described in detail within the next sections.

### Index of microcirculatory resistance measurement

IMR was measured using thermodilution technique on the CoroFlow system (Coroventis, Uppsala Sweden) as previously described, immediately before stenting and at completion of pPCI [4]. Briefly, a standard pressure wire (PressureWire X, Abbott, Santa Clara, CA) was calibrated, equalized and advanced towards the distal third of the IRA. After intracoronary injection of 250 μg isosorbide dinitrate, mean aortic pressure (Pa), mean distal pressure (Pd) and mean transit time (tTmean) were measured both at baseline and at hyperaemia, achieved with intravenous infusion of adenosine at a rate of 140 µg/kg/min. Mean transit time was calculated as the average of three transit time measurements during three separate injections of 3 ml of room temperature 0.9% saline solution. IMR was then calculated as follows:

$$IMR \, = \, Pd_{(hyperaemia)} \times \, tTmean_{(hyperaemia)}$$

when assessed before stenting, IMR was measured either according to the above formula and also corrected for coronary wedge pressure, to account for residual collateral flow:

$$IMR = {\text{Pa}}\left( {{\text{hyperaemia}}} \right){ } \times {\text{ tTmean}}\left( {{\text{hyperaemia}}} \right){ } \times \frac{{Pd\left( {hyperaemia} \right) - Pcorwedge}}{{Pa\left( {hyperaemia} \right) - Pcorwedge}}$$

Coronary wedge pressure was measured by the pressure sensor of the pressure-wire during prolonged angioplasty-balloon inflation.

In a subset of patients, IMR was measured also in one of the two non-IRAs. The selection of which non-IRA to assess was left to operator’s discretion.

### Quantitative flow ratio measurement

At the same time points when IMR was measured, and only when measurement of IMR was completed, angiographic images were acquired at 15 frame/second with manual injection of contrast dye during maximal hyperaemia, using a monoplane radiographic system (Siemens Healthcare, Germany). Pre-specified projections were agreed with the radiographer to guarantee views at least 25° apart.

Three-dimensional quantitative coronary angiography (3D-QCA) and then QFR were measured off-line using QAngio® XA 3D software (Medis, Leiden, the Netherlands) by two independent operators (RS, MS) blinded to clinical, IMR and CMR data. Contrast-flow QFR (cQFR) and fixed-flow QFR (fQFR) were provided. Cases of disagreement were resolved by consensus.

Since IMR is measured during maximal hyperaemia, we elected to assess IMR_angio_ under hyperaemic conditions, as well. For this reason, QFR was assessed using the angiographic views taken at peak hyperaemia during adenosine infusion. Pressure-wire was left in place during angiographic acquisition to allow calculation of QFR exactly at the site of the distal pressure/temperature transducer.

As per IMR, in a subset of patients, QFR was measured also in one of the two non-IRAs.

### Angiography-derived index of microcirculatory resistance

IMR_angio_ was derived starting from the formula for calculation of IMR.

$${\text{IMR }} = {\text{ Pd}}_{{({\text{hyperaemia}})}} \times {\text{ tTmean}}_{{({\text{hyperaemia}})}}$$

where Pd_(hyperaemia)_ is distal pressure at hyperaemia and tTmean_(hyperaemia)_ is mean transit time at hyperaemia. By multiplying and dividing by hyperaemic aortic pressure (Pa_(hyperaemia)_), the formula becomes:

$$IMR = Pa\left( {hyperaemia} \right)\, \times \, \frac{{Pd\left( {hyperaemia} \right)}}{{Pa\left( {hyperaemia} \right)}}\, \times \,tTmean\left( {hyperaemia} \right)$$

Since QFR is a surrogate of Pd(hyperaemia)/Pa(hyperaemia) ratio, (QFR ~ $\frac{Pd(hyperaemia)}{Pa\left(hyperaemia\right)}$), QFR can be used to replace $\frac{Pd(hyperaemia)}{Pa\left(hyperaemia\right)}$ in the formula. Similarly, tTmean_(hyperaemia)_ can be expressed as the ratio between the number of frames (Nframes) for contrast dye to travel, during hyperaemia, from the guiding catheter to a distal reference (corresponding to the position of the distal marker of the pressure wire) divided by the acquisition rate (fps).

In this way the formula becomes:

$$IMR{{angio}} = Pa\left( {hyperaemia} \right) \times QFR \times \frac{{Nframes\left( {hyperaemia} \right)}}{fps}$$

being fps set at 15 frame/second for QFR measurement.

IMR_angio_ was derived in the IRA at the same time points when IMR was measured, and in the non-IRAs where IMR assessment was performed per protocol.

### Cardiovascular magnetic resonance imaging

CMR scans were performed at 48 h after pPCI using a 3.0 T scanner (either MAGNETOM TIMTrio or MAGNETOM Verio, Siemens Healthcare, Germany). Sequence acquisition was performed as previously described [11].

Microvascular obstruction (MVO) was defined as hypointense area within the hyperenhancement region on the late gadolinium enhancement images and was manually contoured [11]. We considered an MVO > 1.55% of left ventricle mass as prognostically significant based on de Waha et al. [12].

### Statistical analysis

After verifying normal distribution by Shapiro–Wilk’s test, variables were expressed as mean and ( ±) standard deviation (SD) or as median accompanied by interquartile range (IQR), as appropriate. Frequencies were compared using Chi square test or Fisher’s exact test, as appropriate. Continuous variables were compared using T test or analysis of variance (ANOVA) with Scheffe’s post-hoc comparisons, as appropriate. Non-normally distributed continuous variables were compared using Mann–Whitney’s test or Kruskall Wallis’ test, as appropriate. T test or Wilcoxon test were used as appropriate for paired samples. Correlations between variables were expressed using Pearson r or Spearman rho coefficients as appropriate.

Inter-rater reliability was assessed by interclass coefficient (ICC) and corresponding 95% confidence interval.

The concordance between IMR_angio_ and IMR was assessed by Bland–Altman plot and the diagnostic efficiency of IMR_angio_ in predicting IMR ≥ 40U and MVO > 1.55% was assessed by the area under the receiver-operating characteristic curve. Youden index analysis was used to identify best cut-off of IMR_angio_ for prediction of post-pPCI IMR ≥ 40U.

Statistical analysis was performed using SPSS 24.0 (SPSS, Inc Chicago, Illinois) and a p value < 0.05 was considered statistically significant.

## Results

### Clinical and procedural characteristics

**Fig. 2:**
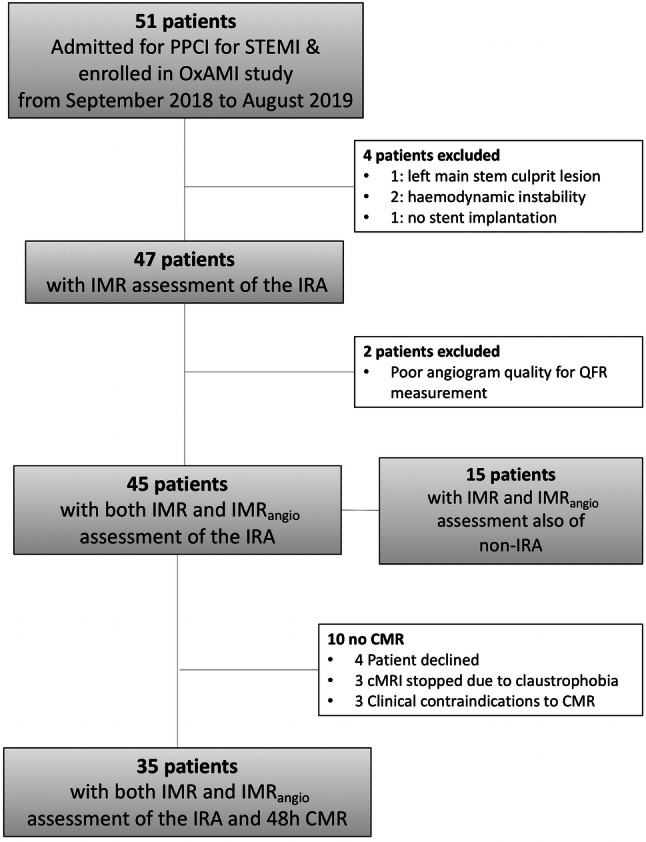
Patients flow chart

A total of 45 STEMI patients were included in the current analysis (Fig. 2). Clinical and procedural characteristics are presented for the whole cohort (Table 1) and stratified according to IMR_angio_ above or below 40 U (Supplementary Table 1 and 2). The cut-off of 40U for IMR_angio_ was derived from ROC analysis (see “Correlations between IMR and IMR_angio_” section).

**Table 1:** Overall clinical, angiographic and procedural characteristics

Clinical data	n = 45
Age, years	61.5 (54.7–71.0)
Male (%)	35 (77.8)
Hypertension (%)	28 (62.2)
Hypercholesterolemia (%)	19 (42.2)
Active Smoker (%)	2 6(57.0)
Diabetes (%)	8 (17.7)
Family history of CAD (%)	14 (31.1)
Ischemic time, minutes *(IQR)*	196.0 (127.5–425.5)
Culprit vessel	
* LAD* (%)	22 (48.8)
* LCx* (%)	6 (13.3)
* RCA* (%)	17 (37.9)
TIMI flow at presentation	
0 (%)	26 (57.8)
1 (%)	4 (8.9)
2 (%)	10 (22.2)
3 (%)	5 (11.1)
Periprocedural medication	
* Aspirin* (%)	45 (100.0)
* Clopidogrel* (%)	45 (100.0)
* Heparin* (%)	21 (46.7)
* Bivalirudin* (%)	24 (53.3)
* GPIIbIIIa inhibitors* (%)	3 (6.6)
Angiographic and procedural data	
Thrombus aspiration (%)	10 (22.2)
Predilation (%)	45 (100)
Total stent length, mm	24.0 (20.0–38.0)
Stent diameter, mm	3.5 (3.0–4.0
Postdilation (%)	38 (84.4)
Final TIMI flow	
0 (%)	0 (0.0)
1 (%)	2 (4.4)
2 (%)	3 (6.7)
3 (%)	40 (88.9)
Thrombus score ≥ 4	23 (51.1)
*Haemodynamics*	
*Pre-stenting*	
Hyperemic Pd/Pa	0.75 (0.61–0.85)
CFR	1.27 (1.11–1.67)
IMR	48.6 (25.5–60.3)
cQFR	0.76 (0.64–0.86)
fQFR	0.74 (0.57–0.84)
IMR_angio_	37.3 (23.7–50.2)
*Post-pPCI*	
Hyperemic Pd/Pa	0.95 (0.90–0.98)
CFR	1.81 (1.51–2.26)
IMR	30.9 (16.5–52.9)
cQFR	0.95 (0.88–0.98)
fQFR	0.95 (0.89–0.99)
IMR_angio_	30.0 (19.3–43.9)

**Table 2:** CMR at 48 h assessment stratified according to post-pPCI IMR_angio_ ≥ 40U

Variable	Post-pPCI IMR_angio_ < 40U	Post-pPCI IMR_angio_ ≥ 40U	p-value
Number of patients	21 (67.7)	10 (32.3)	
LVEDV(ml)	151 (126–179)	166 (146–201)	0.19
LVESV(ml)	80 (56–108)	83 (67–121)	0.67
LVEF(%)	49 (40–54)	50 (41–57)	0.70
Infarct Size(g)	18 (13–27)	22 (15–30)	0.86
Infarct Size(%)	22 (18.0–30)	25 (19–31)	0.77
MVO > 1.55%	4 (19)	6 (60)	0.03

### Correlations between IMR and IMR_angio_

Satisfactory inter-rater reliability was detected for QFR (ICC 0.83 (CI95% 0.61–0.93), F = 6.37, p < 0.001) and IMR_angio_ (ICC 0.93 (CI95% 0.84–0.97), F = 14.02, p < 0.001).

Good correlation was observed between FFR and QFR (Supplementary Fig. 1).

**Fig. 3:**
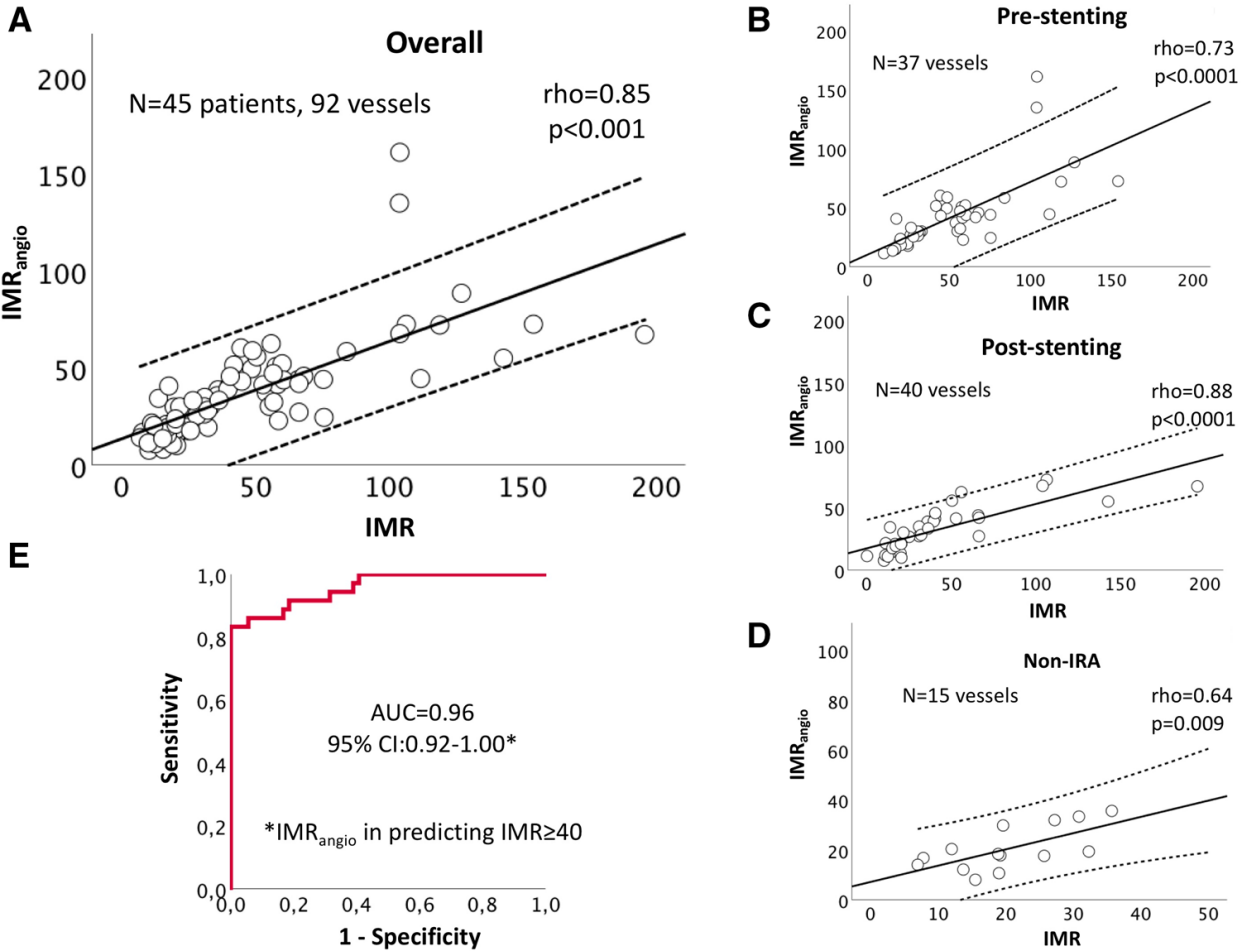
IMR_angio_ and IMR correlations in acute STEMI patients. Scatter plots summarise significant correlations between IMR_angio_ and IMR in the overall cohort of 92 lesions assessed (**a**) and then split into IRA before stent implant (**b**), IRA after stent implant (**c**) and non-IRA (**d**). Dotted lines represent 95% Confidence interval. Panel E reports ROC curve analysis for IMR_angio_ in predicting IMR ≥ 40U in the whole cohort of 92 lesions

IMR and IMR_angio_ were significantly correlated in the overall sample of 92 lesions (37 IRA pre-pPCI, 40 IRA post-pPCI and 15 non IRA) (ρ = 0.85, p < 0.001). Correlation between the two variables was maintained when analysis was restricted to only IRA pre-pPCI (ρ = 0.73, p < 0.001), IRA post-pPCI (ρ = 0.88, p < 0.001) and to the non-IRA (ρ = 0.64, p = 0.009) (Fig. 3).

Pre-pPCI IMR_angio_ was also significantly correlated with pre-pPCI IMR corrected by coronary wedge pressure (ρ = 0.80, p = 0.03).

Notably, both IMR_angio_ and IMR were significantly lower in the non-IRA compared to IRA (IMR_angio_ = 17.8U (12.2–29.9) vs 30.0U (20.5–44.3), p = 0.006; IMR = 19.0U (12.5–27.5) vs 31.0 (16.8–55.2), p = 0.01) (Supplementary Fig. 2).

ROC curve analysis showed an excellent diagnostic performance of IMR_angio_ in predicting an IMR ≥ 40 U (AUC = 0.96 (CI95% 0.92–1.00, p < 0.001; Fig. 3e). The optimal cut-off of IMR_angio_ for prediction of IMR ≥ 40 U was 40U (sensitivity 83.0%, specificity 100%, negative predictive value 90.2%, positive predictive value 96.8%, diagnostic accuracy 92.4%).

**Fig. 4:**
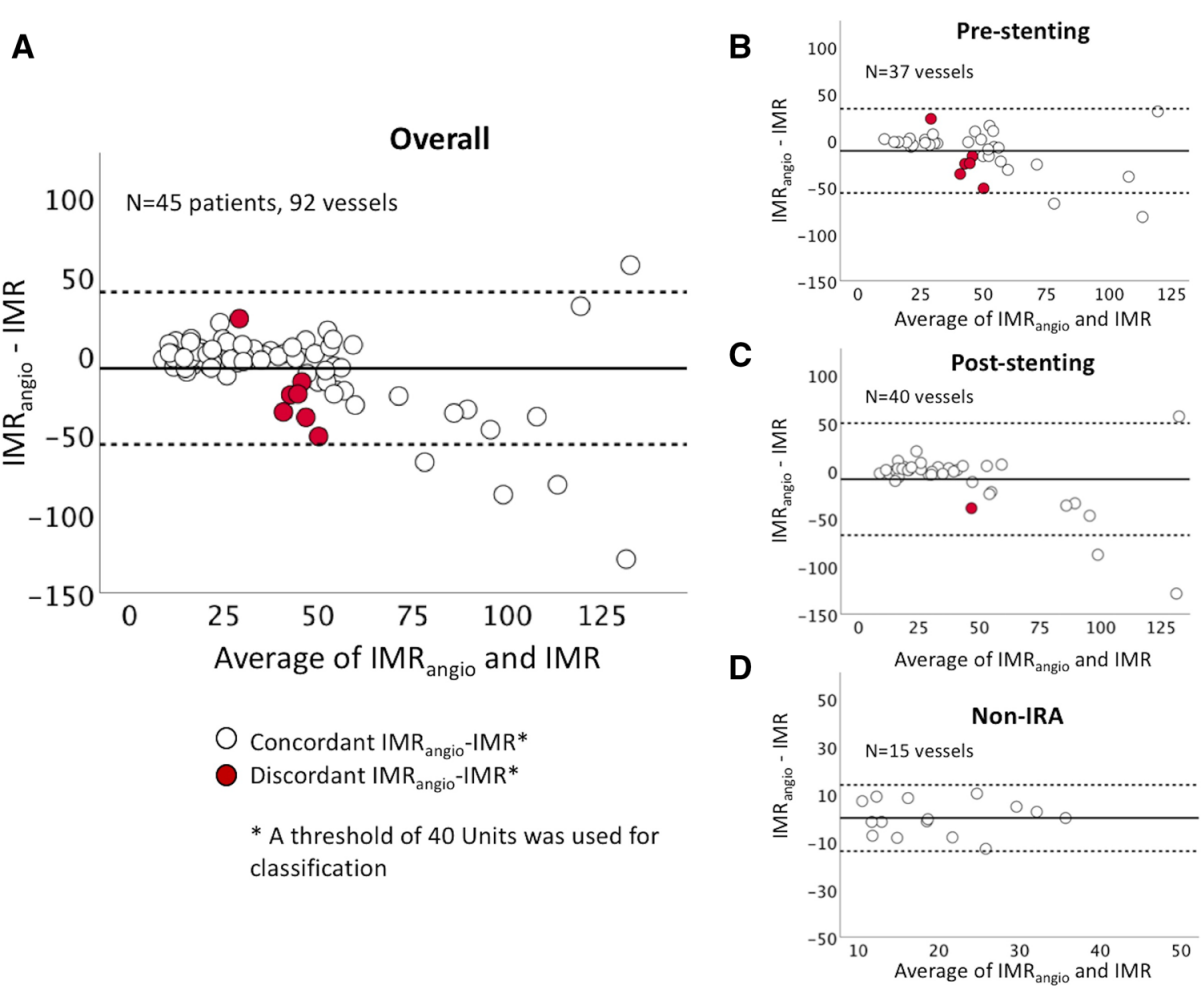
IMR_angio_ and IMR concordance. Bland–Altman plots summarise concordance between IMR_angio_ and IMR in the overall cohort of 92 lesions (**a**) and then split into IRA before stent implant (**b**), IRA after stent implant (**c**) and non-IRA (**d**)

Bland Altman analysis further confirmed concordance between IMR_angio_ within the whole sample and across subgroups (IRA pre-pPCI, IRA post-pPCI and non IRA) (Fig. 4). Only seven discordant cases were identified when a threshold of 40U was applied for both IMR and IMR_angio_. Binary logistic regression analysis could not identify any clinical or procedural factors associated with IMR/IMR_angio_ discordance (Supplementary Table 3).

### Variation of IMR and IMR_angio_ after pPCI

**Fig. 5:**
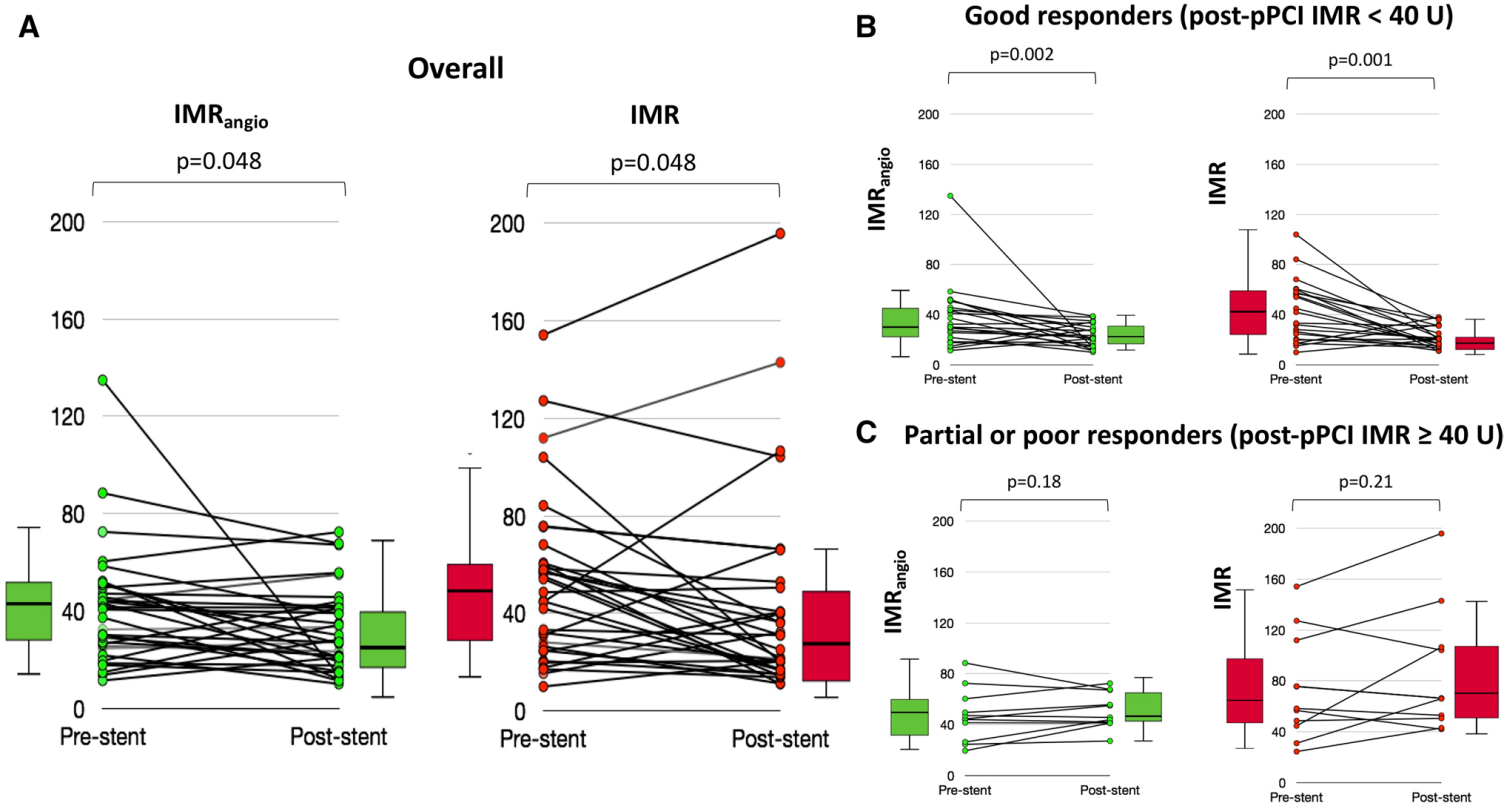
IMR_angio_ and IMR variations before and after stent implant. IMR_angio_ and IMR reduce after stent implantation (**a**). The change in IMR_angio_ consistently mirrored the change in IMR; the relationships persist when patients were divided into ‘good’ (**b**) or ‘partial-poor’ (**c**) responders to stent implant

Assessment of both IMR_angio_ and IMR before and after stenting was available in 33 out of 45 patients. Both IMR_angio_ and IMR decreased significantly after stenting in the IRA (IMR_angio_ from 40.7U (25.0–50.2) to 28.2 (20.2–41.7), p = 0.048; IMR from 48.6U (25.5–64.4) to 31.0 (16.9–51.7), p = 0.048) (Fig. 5). Variation in IMR_angio_ mirrored the one observed for IMR when patients were labelled as good or partial/poor responders to stenting, based on post-pPCI IMR ≥ or < 40U, respectively. In good responders IMR_angio_ went from 32.4U (23.7–48.2) to 21.3U (14.9–31.7) (p = 0.002) and IMR from 41.9U (22.6–58.9) to 20.3U (15.0–28.0) (p = 0.001). In partial/poor responders IMR_angio_ went from 44.3U (25.0–57.6) to 44.8U (41.2–64.3) (p = 0.18) and IMR from 57.5U (34.4–102.8) to 66.2U (43.1–105.9) (p = 0.21). Using the threshold of 40U, post-pPCI IMR categorized 63.6% of patients as good responders, whilst post-pPCI IMR_angio_ categorized 69.7% of patients as good responders (p = 0.69). IMR_angio_ presented a 3% misclassification rate for response to stenting, with only 1 out of the 33 patients misclassified as “good responder” by IMR_angio_ and labelled as “poor responder” according to IMR variation post pPCI.

### Correlation between IMR_angio_ and MVO

CMR data are summarised in Table 2 and stratified according to post-pPCI IMR_angio_ above or below 40U.

**Fig. 6:**
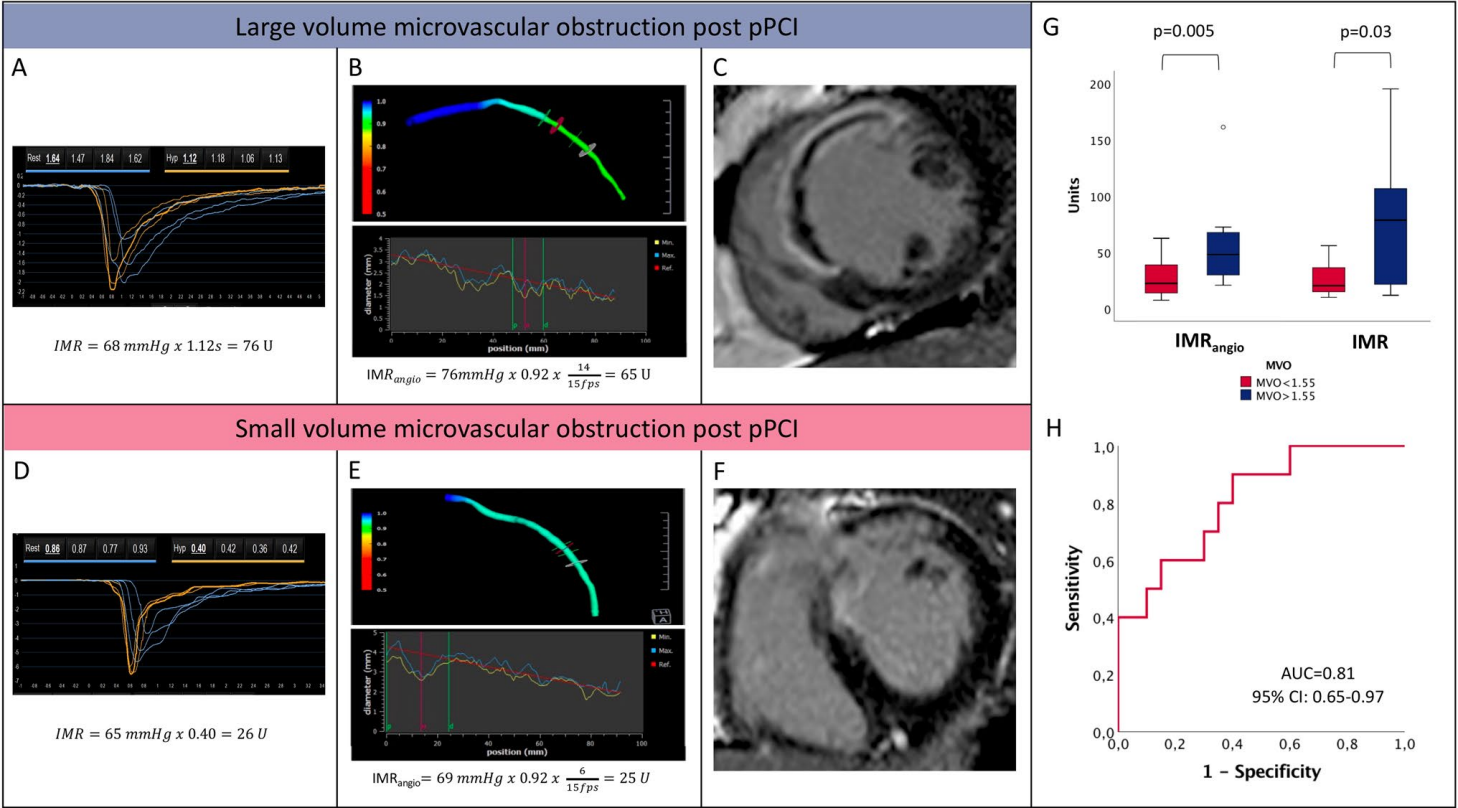
IMR_angio_ and MVO. The figure depicts two STEMI cases with IMR (**a**, **d**), IMR_angio_ (**b**, **e**) assessment and corresponding short axis CMR images with presence (**c**) and absence (**f**) of MVO. The correlation between IMR_angio_ and IMR with the occurrence of clinically relevant MVO (> 1.55% of left ventricle mass) is summarised by the box plots (**g**). Panel H depicts the ROC curve analysis of post-pPCI IMR_angio_ in predicting MVO > 1.55

IMR_angio_ was significantly higher in patients with MVO > 1.55% (48.1U (29.3–68.9) vs 22.6U (13.7–39.0), p = 0.005). Post-pPCI IMR_angio_ presented a satisfactory efficiency for prediction of MVO > 1.55% (AUC = 0.81 (CI95% 0.65–0.97), p = 0.006) (Fig. 6). At the pre-specified cut-off of 40U, IMR_angio_ presented a 60.0% sensitivity, 80.0% specificity, 83.3% negative predictive value, 60.0% positive predictive value and 76.5% diagnostic accuracy).

## Discussion

In the current study, we have derived and validated IMR_angio_ as a novel and pressure-wire-free index for the assessment of coronary microcirculation in STEMI patients. We have specifically observed that:

1. IMR_angio_ is significantly correlated with IMR both in the IRA and in the non-IRA of STEMI patients
2. Both IMR and IMR_angio_ are significantly higher in the IRA than in the non-IRA
3. A value of 40 U appears the best threshold of IMR_angio_ to predict an abnormal IMR (≥ 40 U) in STEMI patients
4. The correlation between IMR_angio_ and IMR is maintained when these variables are measured before or after pPCI
5. IMR_angio_ variation before and after pPCI mirrors the same variation that is observed in IMR
6. IMR_angio_ measured at the end of pPCI is higher in patients with significant MVO and can predict the occurrence of significant MVO (> 1.55% of left ventricle mass).

The availability and performance of pPCI have changed the prognosis for patients presenting with STEMI. However, up to 25–33% of STEMI patients will develop heart failure within five years of treatment, despite contemporary therapy [13]. Extensive coronary microvascular injury results in suboptimal reperfusion and this portends a larger infarct size and a higher risk of adverse remodelling [12]. Identifying, minimising and potentially reversing microvascular injury in STEMI is an unmet clinical need.

**Fig. 7:**
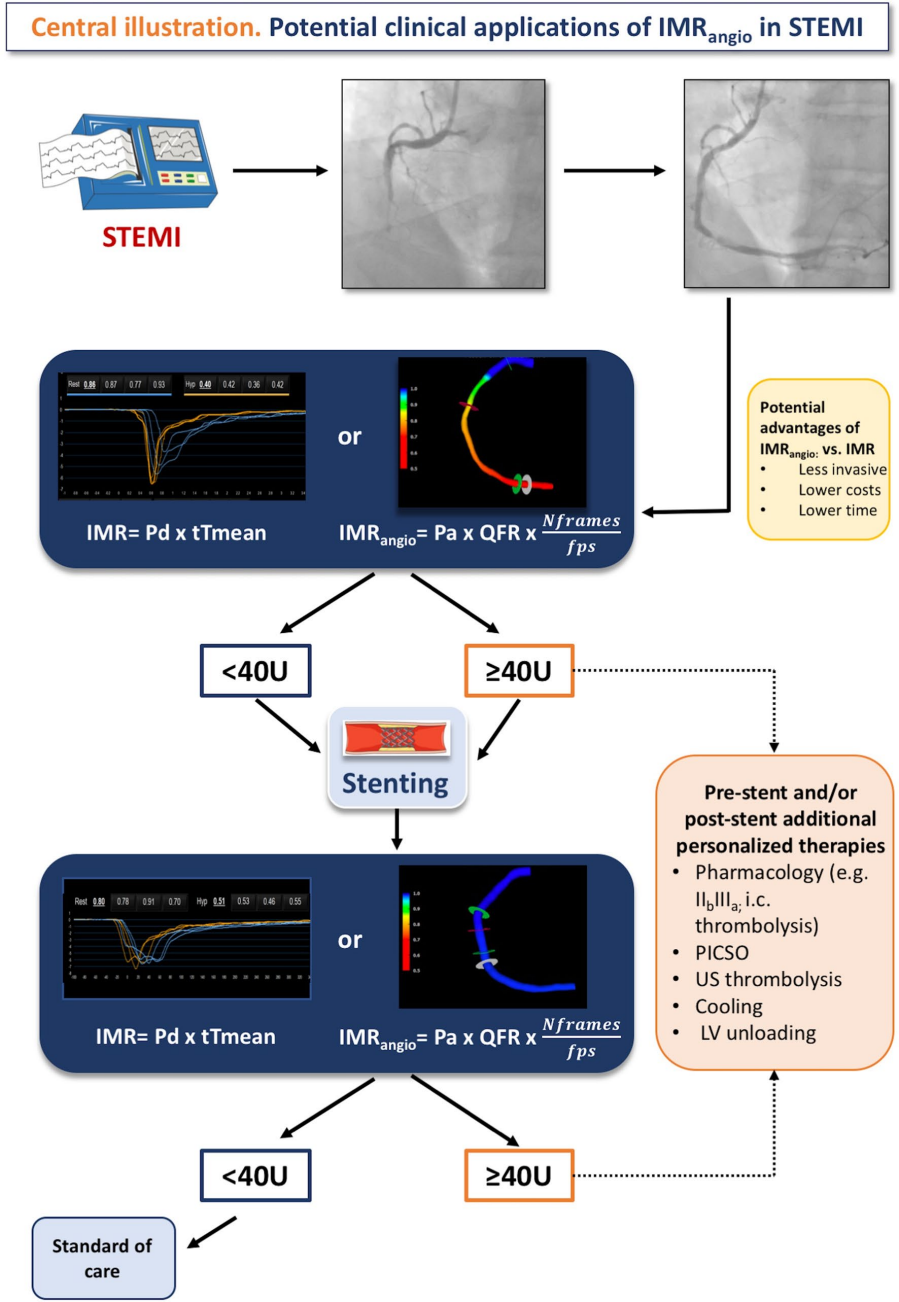
Potential clinical implications of IMR_angio_ in STEMI. IMR or IMR_angio_ can be used to assess microvascular function in patients with STEMI undergoing pPCI before stenting (after flow restoration in the IRA) and at completion of pPCI

In addressing this challenge, assessing the status of coronary microvasculature within the catheter laboratory at the time of STEMI is pivotal since it has the potential to triage patients who might benefit from additional therapy. Early diagnosis/identification of “high risk’ individuals is essential and IMR measurement using pressure-wire can offer a reasonable compromise between practicality and diagnostic accuracy. However, measuring IMR increases procedural time, cost and has an intrinsic (but small) risk related to additional wire manipulation of the IRA (Fig. 7).

Within routine interventional practice, novel angiography-based indices are becoming available to address the limitations of pressure-wire-based measurement of FFR, using computational flow dynamics to model the coronary artery [8]. Amongst these indices, QFR is the one with the largest amount of data cumulated so far [9, 14]. QFR has been used extensively in routine practice to predict FFR, and its application to derive an angiography-based, pressure-wire-free parameter to depict the status of coronary microcirculation is now emerging [15].

This study demonstrates that IMR derived from QFR, labelled as IMR_angio_, can be measured in STEMI patients in the vast majority of cases in a standard catheter laboratory (95.7% of lesions were successfully analysed for QFR and IMR_angio_). Comparisons showed a significant correlation between IMR and IMR_angio_, as confirmed by the ROC curve analysis. Previously, a post-pPCI IMR ≥ 40U has been shown to be prognostically relevant [3] and notably in our data, IMR_angio_ showed a similar upper cut-off of 40U to predict abnormal IMR. When applying this threshold of 40U for both IMR and IMR_angio_, they were concordant in 92% of cases, especially when the assessment was performed at the end of pPCI or in the non-IRA compared to assessment in the IRA before stent implant.

This result is further confirmed by the Bland–Altman analysis showing that IMR_angio_ and IMR are not numerically different for IMR values below 75U. Above 75U, IMR_angio_ can be instead either higher or lower than IMR. This observation emphasises that the absolute numerical values of the two variables are less related in cases of extreme (very high IMR) microvascular dysfunction (Fig. 4). This may reflect the previous suggestion that agreement between QFR and FFR is negatively affected by the presence of severe microvascular impairment [14]. However, even though the difference between IMR and IMR_angio_ values tends to widen with the severity of microvascular impairment, it remains a clinically meaningful concordance between the two measures. Indeed, both IMR and IMR_angio_ measurements are within the adverse range (> 40U) in cases of extreme microvascular dysfunction, with no cases of severely abnormal IMR presenting a normal IMR_angio_ and vice versa. Notably, the few cases of discordance were clustered around the threshold of 40U (Fig. 4).

Interestingly, in the subset of 15 patients with multivessel assessment, IMR_angio_ and IMR were correlated both in the IRA as in the non-IRA. Moreover, both IMR and IMR_angio_ appeared to be significantly higher in the IRA. This is in line with previous observations that microvascular impairment in the non-IRA, when present, is usually not severe and the observed values of IMR are not significantly different from those measured in patients with stable coronary artery disease 16].

In our study IMR and IMR_angio_ were measured at two time points (before and after stenting). We have previously described that, overall, IMR tends to improve after stenting, as a consequence of flow-mediated dilation of the microvascular bed 4]. However, a proportion of patients appear to experience a suboptimal response to stent implant, ending with a final IMR ≥ 40 U as consequence of post-pPCI IMR increase or incomplete reduction below the desired threshold of 40 U 4]. The same trends were observed for IMR_angio_ in this study, with a similar rate of poor or partial responders to stenting when classification was based either on final IMR_angio_ or IMR.

Recently de Waha et al. have reported, in a pooled cohort of 1688 STEMI patients undergoing post-pPCI CMR, that MVO > 1.55% of left ventricle mass was associated with higher rates of mortality and heart failure at one year 12]. In our study, post-pPCI IMR_angio_ appeared significantly elevated in patients with evidence of clinically significant MVO (> 1.55% of left ventricle mass) on CMR. This observation echoes that by McGeoch et al. who reported higher IMR values in STEMI patients with MVO 2].

Notably, whilst IMR and IMR_angio_ were correlated with the presence of MVO, neither of them presented a strong correlation with the extent of MVO and infarct size. This discrepancy is consistent with previous studies 2]. Potential explanations include the difference in the timing of IMR/ IMR_angio_ measurement and CMR scanning and the fact that IMR/ IMR_angio_ provides a functional assessment of coronary microcirculatory injury, whilst CMR an anatomical one 17].

## Limitations

The relatively small sample size represents a limiting factor to keep into account when interpreting the results of the current study.

A second observation is that QFR and IMR_angio_ were both measured offline. This accounts for a small proportion of lesions that had to be discarded for IMR_angio_ assessment because of suboptimal quality of angiographic views.

One of the advocated benefits of QFR in management of patients with stable coronary disease is that accuracy is maintained in predicting FFR, irrespective of the use of adenosine to achieve maximal vasodilation. The so called “contrast-QFR” represents an index that is pressure-wire and adenosine-free [8]. In our study, in order to replicate IMR, QFR (and thus IMR_angio_), was derived from angiographic views acquired at maximal hyperaemia achieved during intravenous adenosine infusion. In fact, the assessment of microvascular function in STEMI appears to be more reliable and consistent at maximal hyperaemia, since it is less prone to the heterogeneity of the same measurements obtained under resting conditions 18].

Whether IMR_angio_ might maintain the same diagnostic accuracy in predicting IMR and MVO also under non-hyperaemic conditions needs to be evaluated in future studies.

## Conclusions

IMR_angio_ is a pressure-wire-free index with the potential to provide an easier and routine assessment of coronary microcirculation in the emergency setting of STEMI. Ultimately, even though further prospective validation is necessary in STEMI and across the spectrum of coronary artery disease, IMR_angio_ can be an easy, quick and cost-effective point-of-care test for routine assessment of microvascular function in the catheter lab with the ultimate goal of facilitating prognostic stratification and early triage of ad-hoc/personalised therapies.

## Electronic supplementary material

Below is the link to the electronic supplementary material.

Supplementary file1 (DOCX 220 kb)

## Abbreviations

CMR: Cardiovascular magnetic resonance
IMR: Index of microcirculatory resistance
IMR_angio_: Angiography-derived index of microcirculatory resistance
IRA: Infarct related artery
IS: Infarct size
MVO: Microvascular obstruction
Pa: Aortic pressure
Pd: Distal pressure
QFR: Quantitative flow ratio
STEMI: ST elevation myocardial infarction
