# Supplementary material for: Angiography-derived index of microcirculatory resistance as a novel, pressure-wire-free tool to assess coronary microcirculation in ST elevation myocardial infarction
Source: Int J Cardiovasc Imaging. 2020 May 14;36(8):1395–406. doi: 10.1007/s10554-020-01831-7 (PMC7381481; doi:10.1007/s10554-020-01831-7)
Supplement: Supplementary file 1 — Supplementary file1 (DOCX 220 kb) [file 10554_2020_1831_MOESM1_ESM.docx]

**SUPPLEMENTARY MATERIAL**

**Angiography-derived index of microcirculatory resistance as a pressure-wire-free tool to assess coronary microcirculation in ST elevation myocardial infarction**

Giovanni Luigi De Maria, Roberto Scarsini, Mayooran Shanmuganathan, Rafail A. Kotronias, Dimitrios Terentes-Printzios, Alessandra Borlotti, Jeremy P. Langrish, Andrew J. Lucking, Robin P. Choudhury, Rajesh Kharbanda, Vanessa M. Ferreira, Oxford Acute Myocardial Infarction (OXAMI) Study Investigators, Keith M. Channon, Hector M. Garcia-Garcia, Adrian P. Banning.

**Supplementary Figure 1. Correlation between QFR and FFR**

Scatter plot in (a) shows the correlation between the hyperaemic thermodilution-derived transit time and the total frame count (TCF) divided by the angiographic acquisition frame rate (TFC/15 fps).

Scatter plot in (b) shows the correlation between distal coronary pressure (Pd) measured with pressure wire and derived using QFR analysis. QFR-based Pd was derived according to the following formula:

FFR=Pd/Pa (1)

FFR ~ QFR (2)

Solving (1) and (2): QFR ~ Pd/Pa (3)

Pd = QFR x Pa (4).

Scatter plot in (c) shows the correlation between QFR and FFR in the study cohort. Red dotted lines identified QFR and FFR conventional cut-offs (≤0.80).


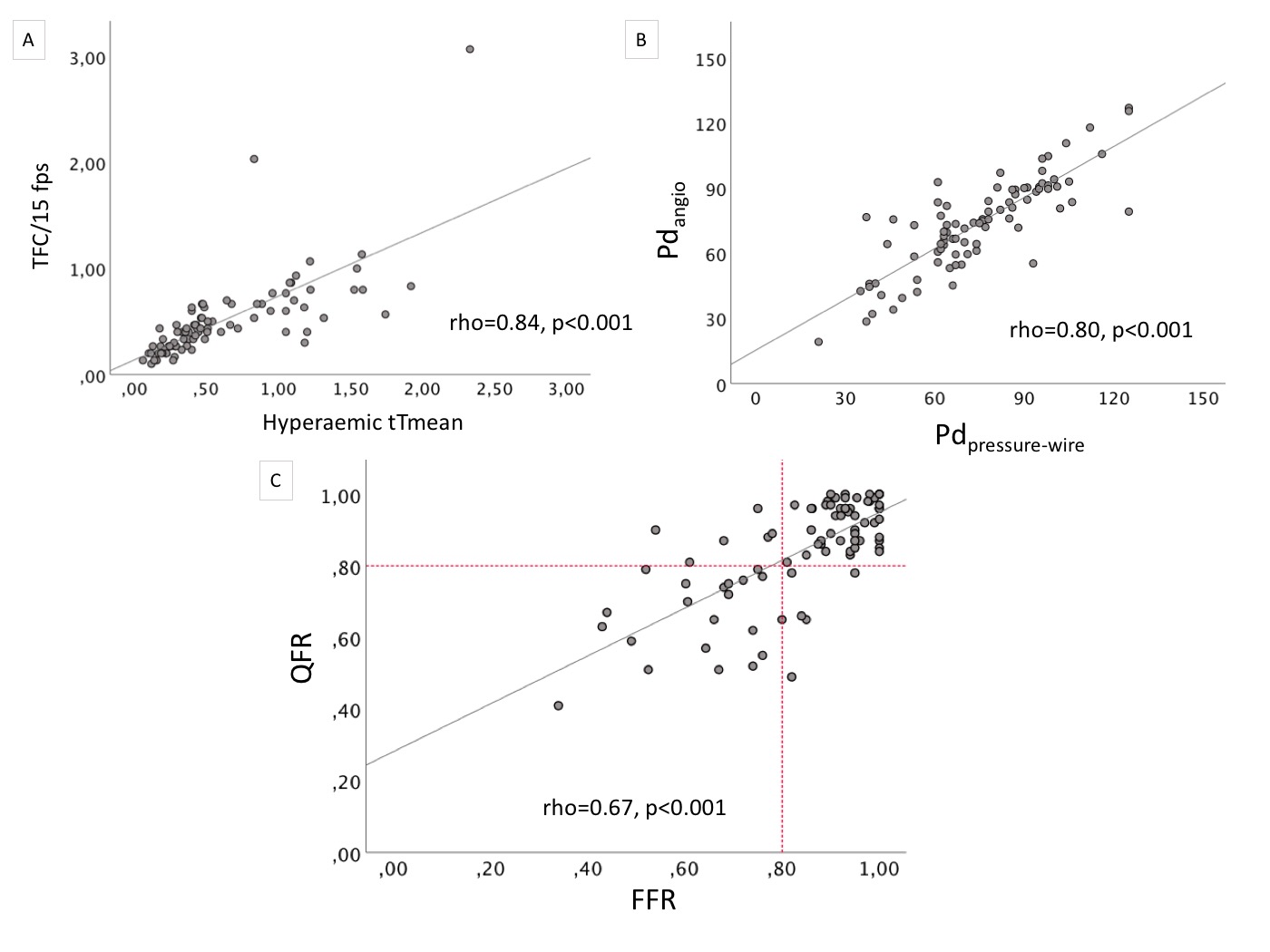


**Supplementary Figure 2. IMR_angio_ and IMR in IRA versus non-IRA.** Box plots show how both IMR_Angio_ (Panel A) and IMR (Panel B) are significantly higher in IRA than in non-IRA. Box plot represent median and interquartile range. Whiskers identify maximum and minimum observations within the upper and lower fences.

**SUPPLEMENTARY TABLES**

| **Supplementary Table 1. Clinical, angiographic and procedural characteristics stratified according to post-pPCI IMRangio≥40U** | | | |
| --- | --- | --- | --- |
| **Variable** | **IMR_angio_<40U** | **IMR_angio_≥40U** | **p-value** |
|  | (N=27) | (N=13) |  |
| **Age**, **years** | 64.0(54.4-69.5) | 57.5(54.2-71.7) | 0.76 |
| **Male** *(%)* | 21(78) | 11(85) | 0.48 |
| **Hypertension** *(%)* | 13(52) | 9(75) | 0.28 |
| **Hypercholesterolemia** *(%)* | 12(48) | 5(42) | 0.50 |
| **Active Smoker** *(%)* | 14(56) | 8(68) | 0.40 |
| **Diabetes** *(%)* | 5(20) | 2(17) | 0.59 |
| **Family history of CAD** *(%)* | 9(36) | 3(25) | 0.71 |
| **Ischemic time, minutes** *(IQR)* | 196.0(128.2-480.5) | 246.0(90.0-614.0) | 0.84 |
| **Angiographic and procedural data** | |  |  |
| **Culprit vessel** |  |  |  |
| *LAD (%)* | 14(52) | 5(38.5) | 0.41 |
| *LCx (%)* | 3(11) | 3(23) |  |
| *RCA (%)* | 10(37) | 5(38.5) |  |
| **TIMI flow at presentation** |  |  |  |
| *0 (%)* | 15(60) | 10(83) | 0.32 |
| *1 (%)* | 2(8) | 0(0) |  |
| *2 (%)* | 4(16) | 2(17) |  |
| *3 (%)* | 4(16) | 0(0) |  |
| **Periprocedural Medication** |  |  |  |
| *Aspirin (%)* | 22(96) | 12(100) | 0.66 |
| *Clopidogrel (%)* | 12(54) | 5(50) | 1.00 |
| *Heparin (%)* | 10(42) | 7(58) | 0.48 |
| *Bivalirudin (%)* | 15(60) | 5(42) | 0.48 |
| *GPIIbIIIa inhibitors (%)* | 0(0) | 3(25) | 0.03 |
| **Predilation** *(%)* | 100 | 100 | 1.00 |
| **Total Stent length, mm** | 24.0(19.0-38.0) | 24.0(20.0-29.5) | 0.75 |
| **Stent Diameter, mm** | 3.5(3.0-4.0) | 3.2(3.0-3.8) | 0.73 |
| **Postdilation** *(%)* | 23(96) | 10(91) | 0.54 |
| **Final TIMI flow** |  |  |  |
| *0 (%)* | 0(0) | 0(0) | 0.006 |
| *1(%)* | 1(4) | 0(0) |  |
| *2 (%)* | 0(0) | 4(36) |  |
| *3 (%)* | 23(96) | 7(64) |  |
| **Thrombus Score ≥4** | 13(48) | 9(69) | 0.31 |
| **Post-pPCI haemodynamic data** |  |  |  |
| Hyperemic Pd/Pa | 0.94(0.90-0.98) | 0.95(0.91-1.00) | 0.64 |
| IMR | 20.1(14.6-31.0) | 85.1(51.1-115.9) | <0.001 |
| CFR | 1.9(1.5-2.3) | 1.5(1.2-2.5) | 0.12 |
| QFR | 0.95(0.87-0.98) | 0.95(0.89-1.00) | 0.48 |
| IMR_angio_ | 21.5(15.0-30.0) | 55.7(42.9-69.9) | <0.001 |
| CAD, coronary artery disease; CFR, coronary flow reserve; IMR, index microcirculatory resistance; IMR_angio_, angiography-derived index of microcirculatory resistance; QFR, quantitative flow reserve; TIMI, thrombolysis in myocardial infarction. | | | |

| **Supplementary Table 2. Clinical, angiographic and procedural characteristics stratified according to pre-stenting IMR_angio_≥40U** | | | |
| --- | --- | --- | --- |
| **Variable** | **IMR_angio_<40U** | **IMR_angio_≥40U** | **p-value** |
|  | (N=19) | (N=18) |  |
| **Age**, **years** | 63.0(55.7-67.2) | 64.5(53.7-71.7) | 0.57 |
| **Male** *(%)* | 13(68.4) | 14(77.8) | 0.71 |
| **Hypertension** *(%)* | 9(47.5) | 11(61.1) | 0.31 |
| **Hypercholesterolemia** *(%)* | 9(47.5) | 7(38.8) | 0.74 |
| **Active Smoker** *(%)* | 13(68.4) | 8(44.4) | 0.29 |
| **Diabetes** *(%)* | 4(21.0) | 3(16.6) | 0.57 |
| **Family history of CAD** *(%)* | 4(21.0) | 5(27.7) | 0.43 |
| **Ischemic time, minutes** *(IQR)* | 170.0(102.5-299.5) | 309.0(193.5-624.0) | 0.03 |
| **Angiographic and procedural data** | |  |  |
| **Culprit vessel** |  |  |  |
| *LAD (%)* | 10(52.6) | 9(50.0) | 0.52 |
| *LCx (%)* | 3(15.8) | 1(5.6) |  |
| *RCA (%)* | 6(31.6) | 8(44.4) |  |
| **TIMI flow at presentation** |  | | |
| *0 (%)* | 12(63.2) | 10(55.6) | 0.96 |
| *1 (%)* | 2(10.5) | 2(11.1) |  |
| *2 (%)* | 4(21.1) | 5(27.8) |  |
| *3 (%)* | 1(5.3) | 1(5.6) |  |
| **Periprocedural Medication** |  | | |
| *Aspirin (%)* | 37(100) | 37(100) | 1.00 |
| *Clopidogrel (%)* | 37(100) | 37(100) | 1.00 |
| *Heparin (%)* | 7(36.8) | 9(50.0) | 0,3 |
| *Bivalirudin (%)* | 12(63.2) | 9(50.0) | 0.20 |
| *GPIIbIIIa inhibitors (%)* | 0(0.0) | 2(5.4) | 0.21 |
| **Predilation** *(%)* | 37(100) | 37(100) | 1.00 |
| **Total Stent length, mm** | 24.0(20.0-38.0) | 23.5(16.5-20.5) | 0.25 |
| **Stent Diameter, mm** | 3.5(3.0-4.0) | 3.5(3.0-3.8) | 0.96 |
| **Postdilation** *(%)* | 18(94.7) | 13(72.2) | 0.44 |
| **Final TIMI flow** |  |  |  |
| *0 (%)* | 0(0.0) | 0(0.0) | 0.04 |
| *1(%)* | 0(0.0) | 2(11.1) |  |
| *2 (%)* | 0(0.0) | 2(11.1) |  |
| *3 (%)* | 19(100) | 14(77.8) |  |
| **Thrombus Score ≥4** | 10(52.6) | 10(55.6) | 1.00 |
| **Pre-stenting haemodynamic data** | |  |  |
| **Hyperemic Pd/Pa** | 0.74(0.61-0.82) | 0.75(0.60-0.88) | 0.52 |
| **IMR** | 26.6(19.7-54.1) | 59.2(47.7-89.1) | <0.001 |
| **CFR** | 1.27(1.06-1.95) | 1.26(1.11-1.53) | 0.71 |
| **QFR** | 0.72(0.55-0.870 | 0.77(0.69-0.86) | 0.30 |
| **IMR_angio_** | 24.5(17.7-30.1) | 50.2(44.0-59.2) | <0.001 |
| CAD, coronary artery disease; CFR, coronary flow reserve; IMR, index microcirculatory resistance; IMR_angio_, angiography-derived index of microcirculatory resistance; QFR, quantitative flow reserve; TIMI, thrombolysis in myocardial infarction. | | | |

| **Supplementary Table 3. Predictors of IMR/IMR_angio_ disagreement** | | |
| --- | --- | --- |
| **Variable** | **OR (95% CI)** | **p-value** |
| Age, years | 1.01 (0.92-1.11) | 0.84 |
| Sex male | 0.67 (0.07-6.47) | 0.73 |
| Smoker | 0.36 (0.05-2.44) | 0.30 |
| Hypertension | 0.41 (0.06-2.73) | 0.35 |
| Diabetes | 1.07 (0.10-11.13) | 0.95 |
| Pain time | 0.99 (0.99-1.00) | 0.39 |
| LAD vs. non LAD | 2.20 (0.33-14.79) | 0.42 |
| Thrombus score | 0.44 (0.08-2.53) | 0.36 |
| TIMI flow | 0.93 (0.37-2.32) | 0.88 |
| MVO | 0.75 (0.41-1.37) | 0.34 |
| IMR value | 1.00 (0.98-1.03) | 0.88 |
| LAD, left anterior descending artery; IMR, index of microcirculatory resistance; IMR_angio_, angiography-derived index of microcirculatory resistance; MVO, microvascular obstruction; TIMI, thrombolysis in myocardial infarction. | | |
